# Identification of HuR–RNA Interfering Compounds by Dynamic Combinatorial Chemistry and Fluorescence Polarization

**DOI:** 10.1021/acsmedchemlett.3c00303

**Published:** 2023-10-06

**Authors:** Serena Della Volpe, Roberta Listro, Francesca Alessandra Ambrosio, Martina Garbagnoli, Pasquale Linciano, Daniela Rossi, Giosuè Costa, Stefano Alcaro, Francesca Vasile, Anna K. H. Hirsch, Simona Collina

**Affiliations:** † Department of Drug Sciences, University of Pavia, Via Taramelli 12, 27100 Pavia, Italy; ‡ Helmholtz Institute for Pharmaceutical Research Saarland (HIPS), Helmholtz Centre for Infection Research (HZI), Campus E8.1, 66123 Saarbrücken, Germany; § Department of Experimental and Clinical Medicine, University “Magna Græcia” of Catanzaro, Campus “S. Venuta”, Viale Europa, 88100 Catanzaro, Italy; ∥ Department of Health Sciences, University “Magna Græcia” of Catanzaro, Viale Europa, 88100 Catanzaro, Italy; ⊥ Net4Science Academic Spin-Off, University “Magna Græcia” of Catanzaro, Campus “S. Venuta”, Viale Europa, 88100 Catanzaro, Italy; # Department of Chemistry, University of Milan, Via Golgi 19, 20133 Milano, Italy; ∇ Department of Pharmacy, Saarland University, Campus E8.1, 66123 Saarbrücken, Germany

**Keywords:** ELAVs, HuR, pt-DCC, STD NMR, Molecular modeling, Fluorescence polarization, Hit identification

## Abstract

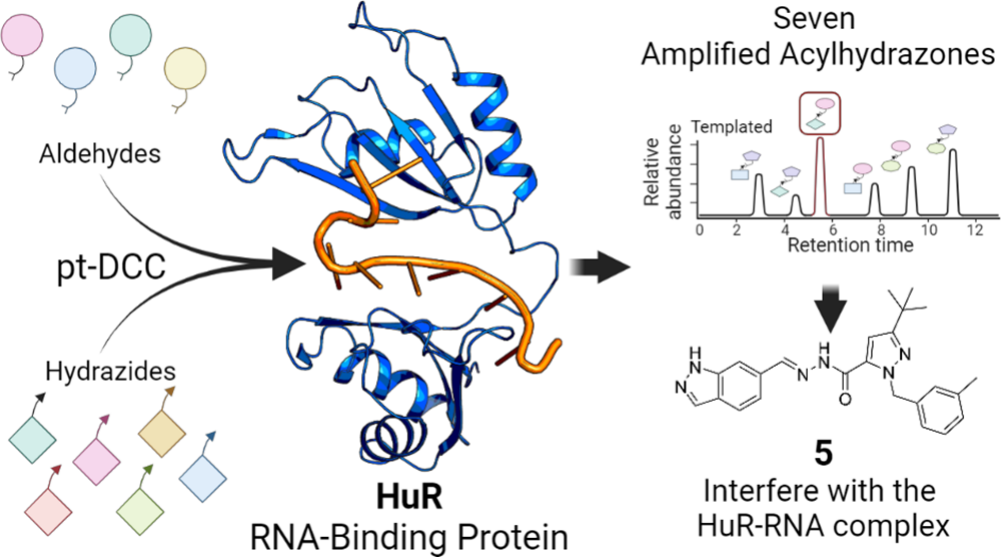

The RNA binding protein HuR regulates the post-transcriptional process of different oncogenes and tumor suppressor genes, and its dysregulation is linked with cancer. Thus, modulating the complex HuR–RNA represents a promising anticancer strategy. To search for novel HuR ligands able to interfere with the HuR–RNA complex, the protein-templated dynamic combinatorial chemistry (pt-DCC) method was utilized. The recombinant RRM1+2 protein construct, which contains essential domains for ligand–HuR binding and exhibits enhanced solubility and stability compared to the native protein, was used for pt-DCC. Seven acylhydrazones with over 80% amplification were identified. The binding of the fragments to HuR extracted from DCC was validated using STD-NMR, and molecular modeling studies revealed the ability of the compounds to bind HuR at the mRNA binding pocket. Notably, three compounds effectively interfered with HuR–RNA binding in fluorescence polarization studies, suggesting their potential as foundational compounds for developing anticancer HuR–RNA interfering agents.

RNA binding proteins (RBPs) play a pivotal role in regulating RNA metabolism and a relevant role in the post-transcriptional process of gene regulation and expression. Dysregulations of RBPs can lead to different pathologies, including neurodegeneration, cardiovascular diseases, and cancer. Consequently, RBPs are considered potential targets for the development of innovative therapeutics.^1,2^ Specifically in the context of cancer, numerous studies have indicated that RBPs are overexpressed across various types of tumors and that the complexes formed by RBPs and RNA are critical for tumor progression. Among the RBPs, the protein HuR, a member of the embryonic lethal abnormal visual (ELAV) protein family, has been identified as a key regulator in multiple facets of tumorigenesis, including cell proliferation, angiogenesis, immune response, and metastasis.^3−5^ Predominantly localized within the nucleus, HuR is involved in post-transcriptional processes including splicing and alternative polyadenylation. Moreover, it can shuttle to the cytoplasm, where it determines the fate of target mRNAs. Therefore, overexpression of HuR and its cytoplasmic accumulation have been associated with various types of cancer, as evidenced by the inhibition of tumor growth in HuR knockout cancer cells.^6−10^ In human cancer cells, HuR plays a role in enhancing the stability of mRNAs associated with proto-oncogenes, transcription factors, cytokines, and growth factors, including TGF-β, c-Fos, COX-2, VEGF, and Bcl-2, at the 3′ extremities (UTRs), thus promoting their expression and contributing to carcinogenesis.^1,11−15^ Equally important, HuR also exerts control over the stress response, the formation of autophagy, the organization of the cytoskeleton, and the expression of pro-inflammatory cytokines and fusogenic proteins.^16,17^

Taken together, these findings suggest that the development of HuR–RNA interfering compounds may pave the way for the discovery of new anticancer agents with a novel mechanism of action. Previous medicinal chemistry efforts have resulted in the identification of HuR ligands (Figure 1) by combining different approaches (*in silico* studies, STD NMR, virtual screening, and fragment-based drug discovery).^18−20^

**Figure 1:**
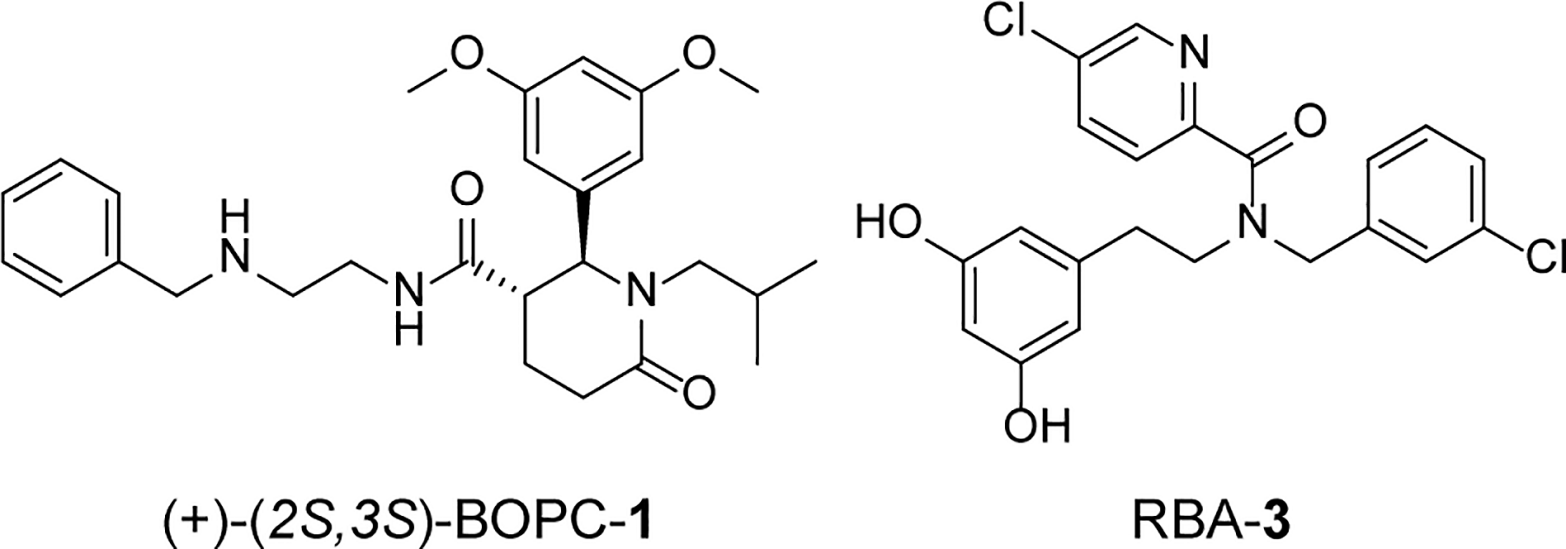
Structures of previously identified HuR ligands.

In this study, we report the identification of novel HuR–RNA complex interfering compounds utilizing the protein-templated dynamic combinatorial chemistry (pt-DCC) approach, since this strategy has been established as a potent tool for discovering new ligands for biological targets.^21^ The pt-DCC approach successfully combines library synthesis and hit identification in a single step. Briefly, through the reversible combination of building blocks, a dynamic combinatorial library (DCL) composed of interchanging products is generated.^22−24^ As the interaction between building blocks is reversible, the distribution of products is influenced by the thermodynamic stability of the resulting compounds. Consequently, the DCL can be sensitive to external signals, such as the introduction of a specific target molecule. Those members of the DCL that exhibit the highest binding affinity for the target are removed from the equilibrium, leading to a subsequent re-establishment of equilibrium within the library.^24^ As a result, the best binders are amplified and can be identified directly from the mixture (Figure 2).

**Figure 2:**
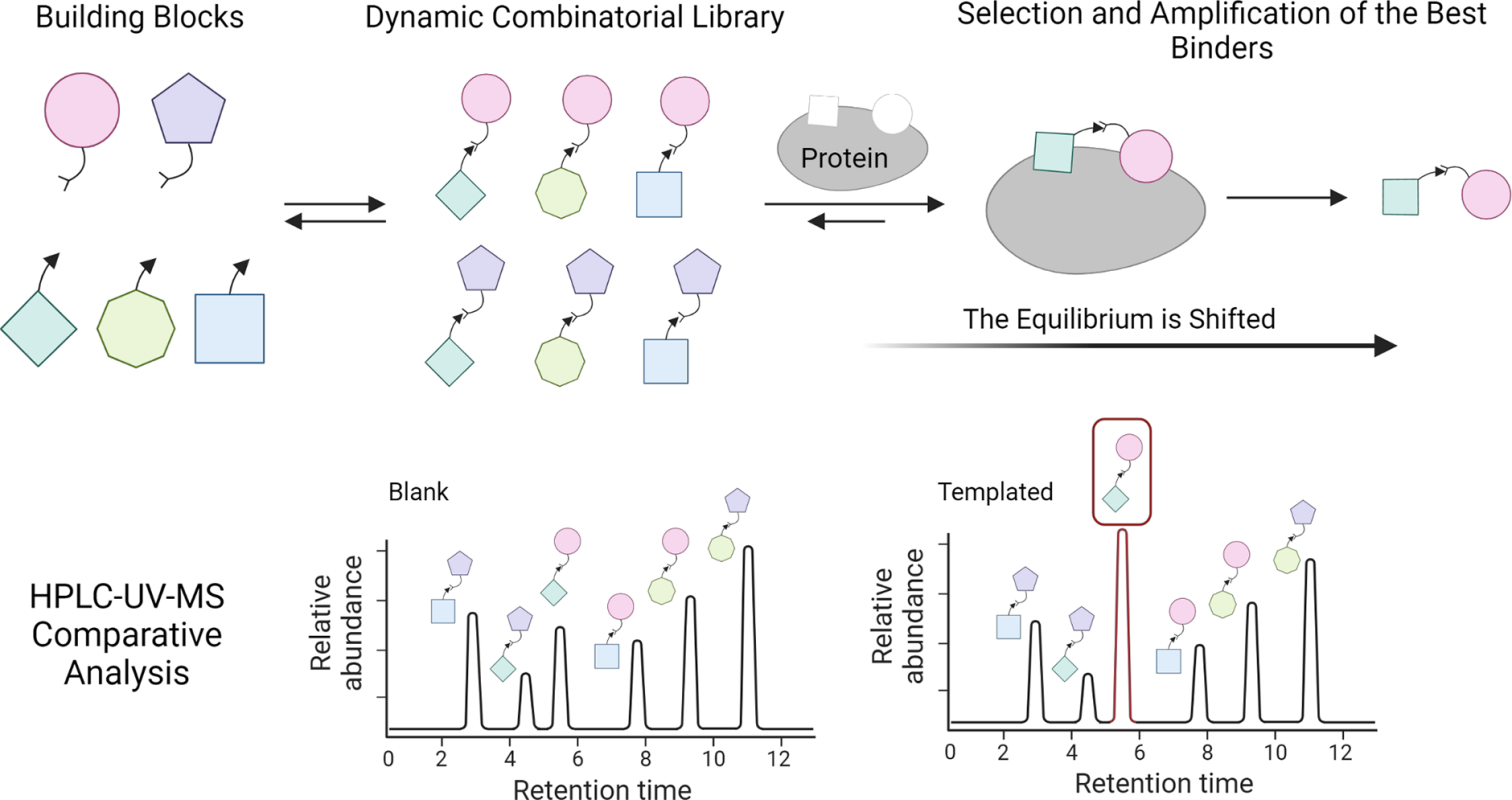
Description of protein-templated DCC. Among the members of a DCL, those that exhibit interactions with a specific protein will undergo amplification compared with the other library members. This amplification can be quantitatively observed using a suitable biophysical method, such as high-performance liquid chromatography with ultraviolet detection (HPLC-UV). Furthermore, the resultant product can be unambiguously identified by coupling the HPLC-UV system with a mass spectrometry (MS) detector.

HuR is structured with three RNA recognition motif (RRM) domains, specifically designated as RRM1, RRM2, and RRM3 (Figure 3). The first two domains, RRM1 and RRM2, are positioned in tandem and are mainly involved in the interaction of HuR with adenine- and uracil-rich elements (AREs) in mRNAs. RRM3, located distally from RRM2 and connected by a basic hinge moiety, contributes to the binding of HuR to the poly(A) tails of target mRNAs and is involved in the oligomerization of HuR.^25−27^

**Figure 3:**
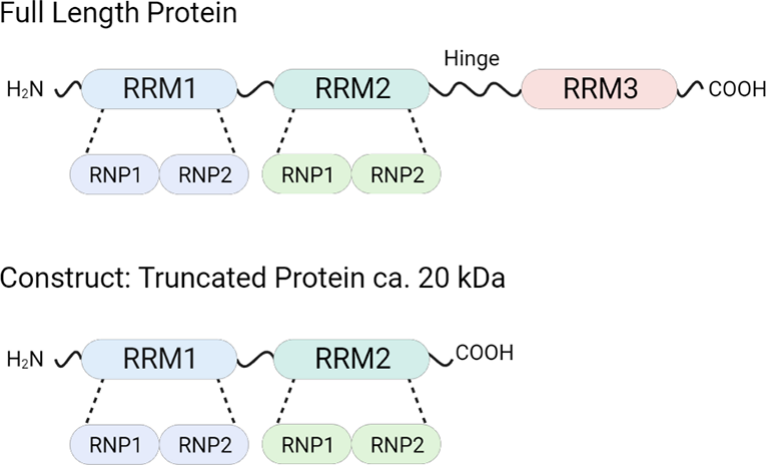
Schematic representation of the full-length protein and truncated protein.

In this study, we used a recombinant approach to express a construct with glycine–glycine–serine (GGS) repeats in order to increase the solubility and stability, instead of the full-length protein.^28^ A recombinant RRM1+2 protein (1–186, ∼20 kDa), generously provided by Sattler from the Bavarian NMR Centre, School of Natural Sciences, University of Munich, was used. This protein contains the two necessary RRM1 and RRM2 domains for ligand–HuR binding and is more soluble, more stable, and less susceptible to aggregation and precipitation than the native protein.

Specifically, we utilized a thermal shift assay (TSA) to test the ability of the RRM1+2 domains to establish initial contact with target mRNAs. We then assessed the stability of the recombinant RRM1+2 construct in various buffers and pH values (10–50 mM; pH range 5–7.5; 150 mM NaCl) to determine the optimal experimental conditions for the pt-DCC experiments.

It is worth noting that pt-DCC experiments can result in artifacts, which may arise, for instance, from the precipitation of the DCL compounds or protein, leading to misleading outcomes resulting in undesired alterations in the equilibrium of the system.

These shifts can depend on numerous variables, including the pH, temperature, solubility, and stability of the components. To track the protein’s stability over time, the melting temperature (*T*_m_) was measured using TSA.^21^ Based on the results obtained, acetate or phosphate buffers were selected for pt-DCC.

For the execution of pt-DCC experiments, we opted for the synthesis of *N*-acylhydrazones, which involves the combination of aldehyde and hydrazide building blocks. This process can be conducted in water, thereby enhancing biocompatibility. However, it is important to note that the formation and exchange of hydrazones are dependent on the buffer and pH utilized. Furthermore, under physiological conditions, such as room temperature and/or neutral pH, these processes are considerably slow, while at acidic pH, equilibrium is rapidly achieved. Based on the past experience of the research group,^29^ considering the building block availability^30^ and the biological target, we built a fragment-inspired DCL library containing four aldehydes and 12 hydrazides (Figure 4) and used it for pt-DCC (Figure 4). The RRM1+2 construct was employed at 40 μM concentration, and to achieve the equilibrium at the chosen pH, 1 mM aniline was added (Table SI-2). The amplified acylhydrazones formed were identified via comparative analysis through HPLC-UV-MS. To promote protein precipitation, acetonitrile was added, and the reaction was brought to a “frozen” state by adjusting the pH.

**Figure 4:**
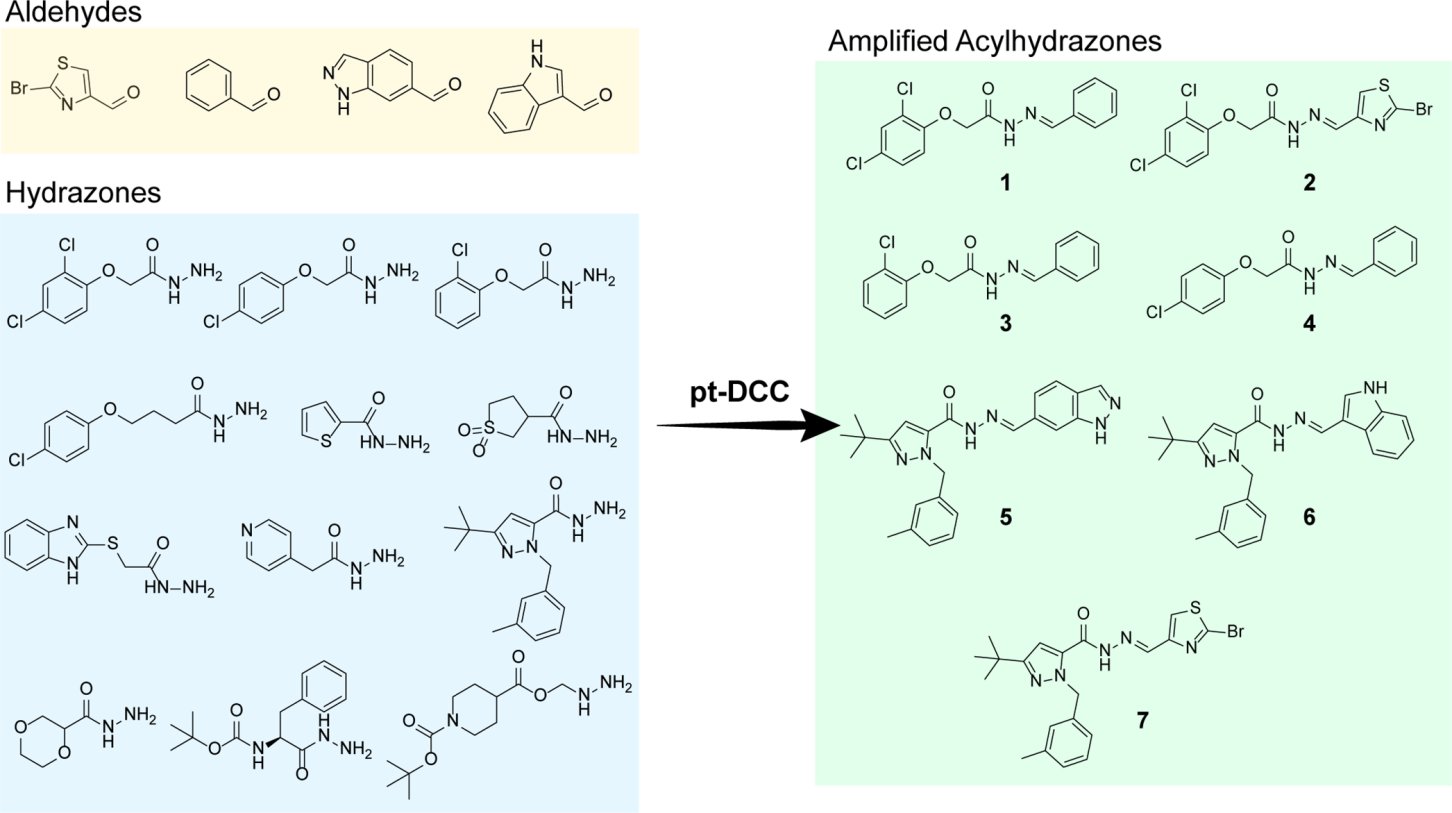
Fragment-inspired DCC libraries adopted by the pt-DCC experiment and amplified acylhydrazones identified.

In Figure 5 an example of screening results is reported. Separation, identification, and quantification of the DCL members represent a key point of the technique, without incurring in any artifacts and determining both equilibrium and pt-amplification. Since the equilibrium was reached after 10 and 12 h in acetate and phosphate buffers, respectively, the amplification was measured at 10 h in acetate buffer or 12 h in phosphate buffer and then at 24 and 30 h in both assays. For the reaction conducted in acetate buffer, significant values were detected at 10 and 24 h for compound **2**: amplification 83% (10 h), 107% (24 h); retention time = 12.86 min (reported by a blue frame).

**Figure 5:**
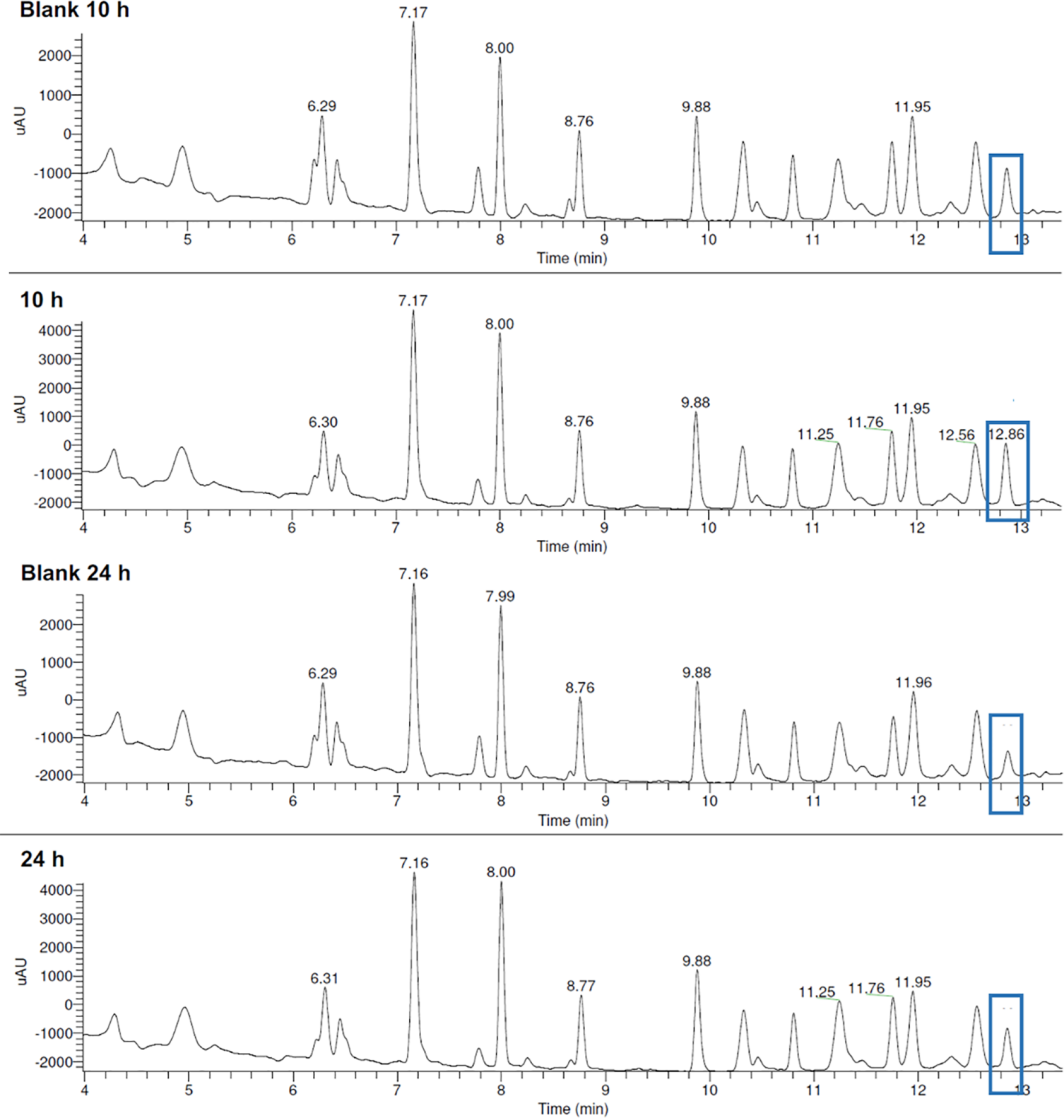
HPLC-UV profiles. Identification of compound **2** is highlighted in blue.

The pt-DCC experiments led to the identification of seven acylhydrazones (with amplification > 80%), as shown in Figure 4. Based on these results, the seven hits that emerged from DCC experiments were selected for synthesis. The compounds were prepared by reacting acylhydrazides and aldehydes in refluxing anhydrous methanol overnight (Scheme 1). All of the compounds were obtained in sufficient amounts and purity for further investigation.

**Scheme 1:**
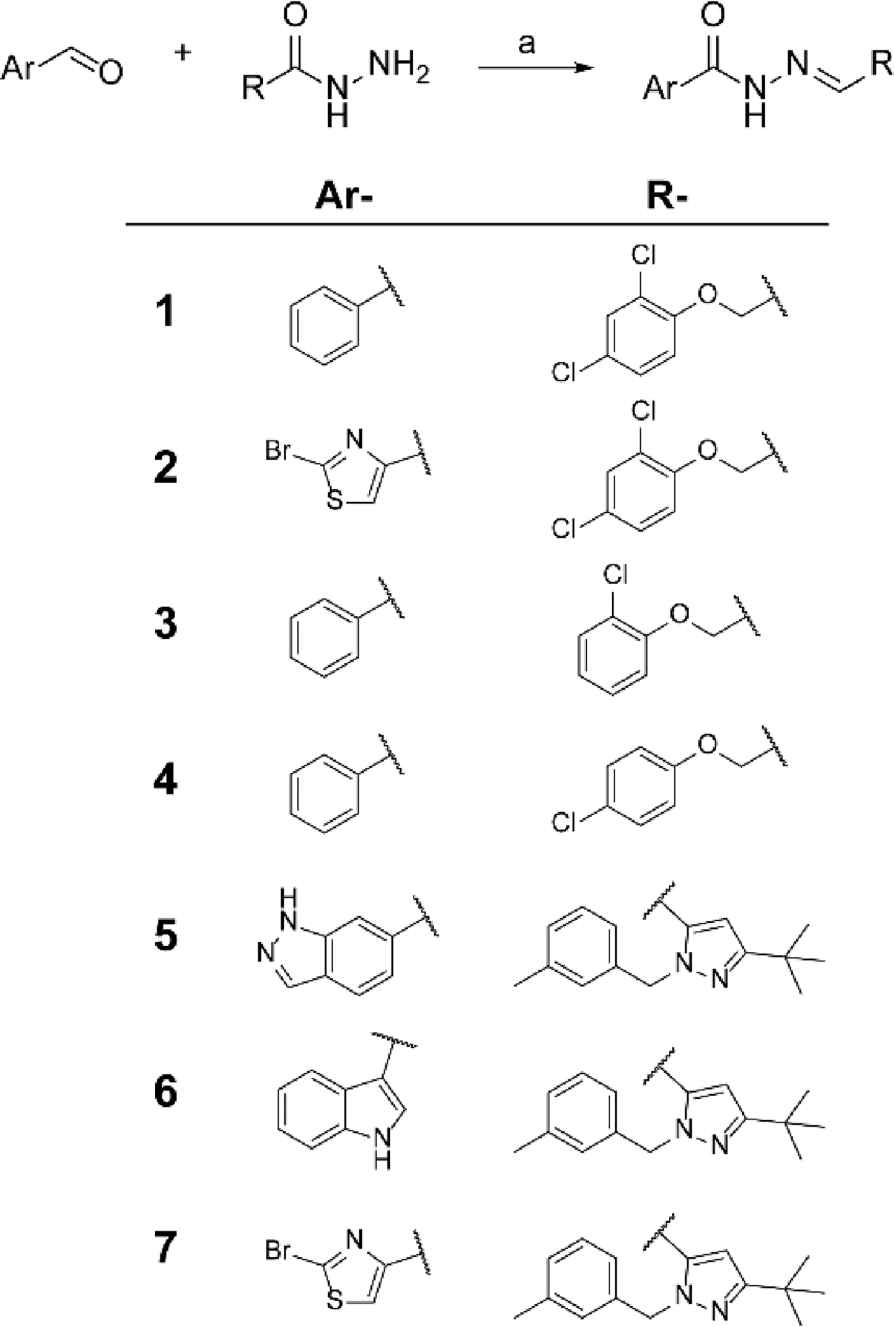
Synthesis of the Seven Hits Reagents and conditions: (a) hydrazide (1 equiv), aldehyde (1 equiv), anhydrous methanol, reflux, N_2_, 16 h.

To confirm the binding of the identified acylhydrazones to HuR, we assessed their interaction with the target protein using saturation transfer difference NMR spectroscopy (STD-NMR). Briefly, STD-NMR is a primary NMR technique employed to study ligand–protein interactions, facilitating the identification of the ligand binding epitope.^31−35^ The protein is selectively irradiated in an area where only its frequencies (and not those of the ligand) are present, including the aliphatic protons (CH_3_) of the aliphatic side chains of the amino acids. The magnetization is transferred by spin diffusion to the other protons of the protein and the ligand in the binding site. The ligand is in large excess with respect to the protein concentration, and the free and bound forms are in fast exchange. This equilibrium and the high ligand/protein ratio allow the detection in the final STD monodimensional spectrum of only the signals of the ligand that are in close contact with the protein, thus revealing the ligand protons involved in the interaction.

However, since compounds **1**–**7** did not exhibit suitable solubility for the STD experiment (0.5 mM phosphate buffer, pH 7.4, with 0.5% DMSO), we opted to evaluate the most amplified fragments, namely, benzyl, indole, indazole, and thiazole. In order to avoid the presence of any reactive moiety on the fragments that could affect the interaction with the protein binding site, the corresponding methyl arylcarboxylate esters were synthesized and used in the STD experiments. The STD-NMR results confirmed that methylindole, indazole, and thiazole fragments can bind *per se* the protein with a strong interaction (Figure SI-7). Specifically, for thiazole and methylindole, the interaction was mediated by all of the protons of the aromatic moiety and by the methoxy group of the ester. For the indazole fragment, the interaction is primarily driven by the aromatic moiety, with a stronger intensity for two singlets assigned to the proton near the nitrogen (on the five-membered ring) and to the ester group (no significant interaction was observed for the methoxy group). Conversely, for methylbenzoate, the signals of 1D proton and STD spectra appeared broad, likely due to its very low solubility in the phosphate buffer used for the interaction studies but suggesting interaction only for the aromatic protons of the fragment. Taken together, these results strongly support the contribution of these moieties in the protein-fragment interactions. Further confirmation regarding the ability of the amplified compounds to bind HuR was derived from *in silico* studies. Indeed, molecular modeling results indicated that all of the studied ligands are capable of recognizing and binding HuR in the pocket involved in the interaction with the mRNA strand. Consequently, by occupying the site responsible for the interaction with the mRNA, the ligands could prevent the binding of the mRNA, thus acting as potential HuR–RNA interfering compounds.

Upon analysis of the binding mode of the ligands, it is observed that all of the compounds are well-accommodated in the HuR binding pocket (Figure 6), establishing various types of interactions, such as hydrogen bonds, halogen bonds, and π–π stacking, with the key residues of the protein binding site.

**Figure 6:**
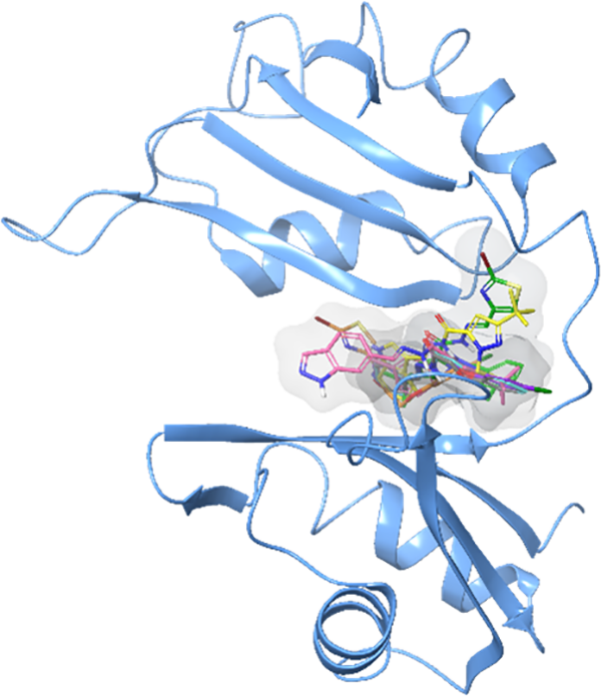
3D representation of acylhydrazone derivatives in complex with the HuR protein. The ligands and the protein are shown as different colored carbon sticks and light-blue cartoon, respectively. The figure was built after the MM-GBSA postdocking analysis.

In detail, compound **5** establishes two hydrogen-bonding interactions, one with Asn25 and one with Arg153, and in addition through the indazole ring is engaged in two π–π stacking interactions with Tyr26 and in hydrogen-bonding interaction with Lys89 (Figure 7).

**Figure 7:**
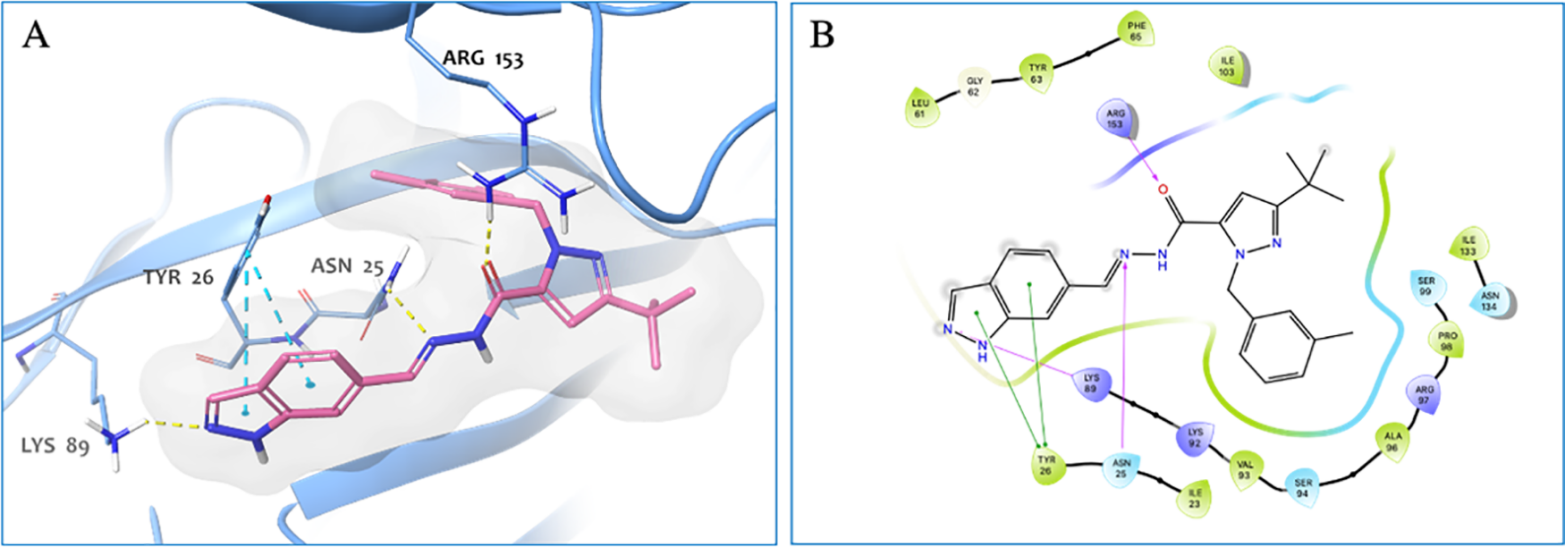
((A) 3D representation of **5** in complex with the HuR protein. The ligand and the protein are shown as pink carbon sticks and light-blue cartoon, respectively. In light-blue carbon sticks the crucial interactions of the protein residues are reported. Hydrogen-bonding and π–π stacking interactions are reported as yellow and cyan dashed lines, respectively. (B) 2D representation of **5** in complex with HuR protein. In the 2D representation, hydrogen-bonding and π–π stacking interactions are shown as magenta and green lines, respectively. The 3D and 2D representations were built after the MM-GBSA postdocking analysis.

Compound **7** is stabilized in the HuR binding pocket, establishing different hydrogen-bonding interactions with residue Arg153. Regarding compound **2**, the Br atom on the thiazole ring establishes different halogen bonds with Ser135 and Arg136; meanwhile, the Cl atom of the phenyl ring forms a halogen bond with Asn25. For compound **6** we notice a hydrogen-bonding interaction between the nitrogen atom of the indole ring and Tyr26. The compound also establishes three hydrogen-bonding contacts with Arg153 and is engaged in a π–π stacking interaction with Phe65. Compounds **1**, **3**, and **4** share a similar binding mode establishing halogen-bonding interactions with Arg97 and/or Ser99 through the Cl atom of the phenyl ring. Moreover, the phenyl ring of each compound is engaged in a π–π stacking interaction with Phe65. The compounds can also form two hydrogen-bonding interactions with Asn25 and Arg153. (The 2D representation of the studied compounds in complex with HuR is reported in the Figure SI-8). Furthermore, all of the compounds are involved in several hydrophobic contacts with the residues of the HuR binding pocket, such as Ile23, Val24, Asn25, Tyr26, Phe65, Lys92, Arg87, Ser99, Arg136, and Arg153. Finally, to evaluate the ligand binding free energy (Δ*G*_bind_) of the acylhydrazone derivatives, MM-GBSA calculations were performed, and the results are reported in Table 1.

**Table 1:** MM-GBSA Δ*G*-Bind values of the acylhydrazone derivatives

compd	MM-GBSA Δ*G*_bind_ (Kcal/Mol)
**5**	–73.35
**7**	–70.95
**2**	–67.74
**6**	–50.26
**1**	–47.32
**3**	–44.41
**4**	–41.21

Lastly, the interfering capacity of compounds **1**–**7**with the HuR–RNA complex was evaluated through the fluorescence polarization (FP) method.^20,36^ A reduction in FP emission upon titration of the HuR–RNA complex with the tested compound is representative of the compound’s ability to destabilize the complex. All of the compounds were tested in a concentration range of 0.049 to 100 μM for a dose–response study. It was not possible to obtain reliable results at higher concentrations due to solubility constraints. The potential of the tested compounds to bind the substrate mRNA was ruled out by evaluating the residual FP emission of mRNA in the presence of the compounds. Epigallocatechin (EGCG) was utilized as a positive control, while DMSO and usnic acid (UA) were employed as negative controls.

Compounds **5**, **7** and **2** were able to prevent the interaction of the protein with mRNA, and in fact, they showed decreased emission comparable to the positive control, in accordance with the *in silico* prediction. As an example, the percent residual FP emission of the best-performing compound, **5**, with EGCG (positive control) and UA (negative control) is reported in Figure 8.

**Figure 8:**
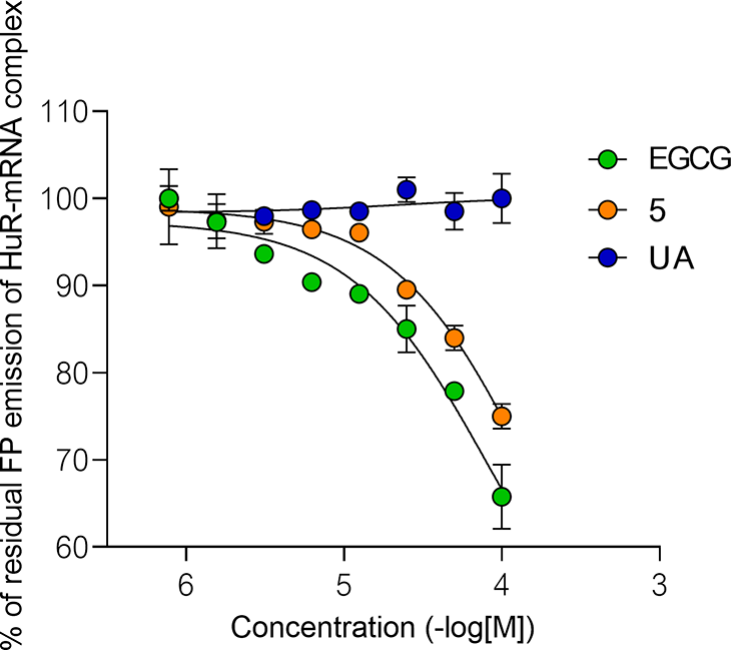
Dose–response curves generated for compound **5**, the positive control EPCG, and the negative control UA in the context of disrupting the binding between HuR and mRNA by using a FP assay. The assay utilized 2.4 μM HuR protein and 45 nM fluorescein-labeled mRNA. For quantifying the results, the % FP value of the HuR–mRNA complex in the presence of DMSO was set as 100% to represent complete complexation, while the % FP value of labeled RNA alone, without any complex formation, was defined as 0%. The experiments were carried out in duplicate, and the data are presented as mean values with the SD indicated to provide an assessment of the data’s variability and precision.

In conclusion, we have reported the first application of pt-DCC for the identification of new HuR–RNA complex interfering compounds. Instead of the native wild-type HuR protein, the more soluble and tractable RRM1+RRM2 construct was employed. As a result of ligand selection, seven hits, characterized by the presence of benzyl, indole, indazole, and thiazole moieties, were amplified. Subsequent STD-NMR experiments confirmed the ability of these fragments to bind HuR, and further *in silico* studies elucidated the binding mode and the interactions established between the ligands and the protein. The amplified hydrazones were then synthesized, and their interfering activity was assessed by FP assays, confirming the ability of compounds **5**, **7**, and **2** to interfere with the HuR–RNA complex. Thus, pt-DCC was successfully applied for the identification of compounds capable not only of interacting with the HuR protein but also of interfering with the formation of the complexes with RNA. In our ongoing efforts to discover potential anticancer agents, the next step in this research will consist of the hit expansion, considering not only the HuR binding affinity but also the physicochemical properties, in particular the solubility.

## Supporting Information Available

Experimental procedures, materials and methods regarding protein expression and purification, pt-DCC experiments, STD-NMR assays, synthesis and characterization of synthesized compounds, fluorescence polarization assays, and molecular modeling (PDF)

## Abbreviations

ARE: adenine- and uracil-rich element
DCL: dynamic combinatorial library
EGCG: epigallocatechin
ELAV: embryonic lethal abnormal visual
FP: fluorescence polarization
MM-GBSA: molecular mechanics generalized Born surface area
pt-DCC: protein-templated dynamic combinatorial chemistry
RBP: RNA binding protein
RRM: RNA recognition motif
STD-NMR: saturation transfer difference NMR spectroscopy
TSA: thermal shift assay
UA: usnic acid
