# Supplementary material for: Identification of HuR–RNA Interfering Compounds by Dynamic Combinatorial Chemistry and Fluorescence Polarization
Source: ACS Med Chem Lett. 2023 Oct 6;14(11):1509–16. doi: 10.1021/acsmedchemlett.3c00303 (PMC10641899; doi:10.1021/acsmedchemlett.3c00303)
Supplement: Supplementary file 1 — ml3c00303_si_001.pdf [file ml3c00303_si_001.pdf]

Identification of HuR–RNA interfering compounds by Dynamic Combinatorial Chemistry and Fluorescence Polarization

Serena Della Volpe#[1,2], Roberta Listro[1], Francesca Alessandra Ambrosio[3], Martina Garbagnoli[1], Pasquale Linciano[1], Daniela Rossi[1], Giosuè Costa [4,5], Stefano Alcaro[4,5], Francesca Vasile[6], Anna K. H. Hirsch*[2,7], Simona Collina*[1].

[1]*University of Pavia, Department of Drug Sciences, Via Taramelli 12, 27100 Pavia (Italy).*
[2]*Helmholtz Institute for Pharmaceutical Research Saarland (HIPS), Helmholtz Centre for Infection Research (HZI), Campus E8.1, 66123 Saarbrücken (Germany).*
[3]*Department of Experimental and Clinical Medicine, University "Magna Græcia" of Catanzaro, Campus "S. Venuta", Viale Europa, 88100 Catanzaro (Italy).*
[4]*Department of Health Sciences, University "Magna Græcia" of Catanzaro, Viale Europa, 88100, Catanzaro (Italy).*
[5]*Net4Science Academic Spin-Off, University "Magna Græcia" of Catanzaro, Campus "S. Venuta", Viale Europa, 88100 Catanzaro (Italy).*
[6]*Department of Chemistry, University of Milan, Via Golgi 19, 20133 Milano (Italy)*
[7]*Department of Pharmacy, Saarland University, Campus E8.1, 66123 Saarbrücken (Germany).*

TABLE OF CONTENT

Material and Methods …SI-3

- Protein expression and purification …SI-3
- Establishment of experimental conditions for the pt-DCC experiment …SI-4
- pt-DCC experiment …SI-6
- STD-NMR assays …SI-12
- Chemistry …SI-15
- Molecular modeling …SI-18
- Fluorescence polarization assays …SI-19
- References …SI-22

TABLES

- Table SI-1. Buffer used for the assessment of pt-DCC. …SI-4
- Table SI-2. Composition of the well for TSA analysis. …SI-4
- Table SI-3. 1H-NMR characterization for compounds 1-7. …SI-16

- Table SI-4. Percentage of residual protein polarization at 100 μM concentration of the tested compounds …SI-20

FIGURES

- Figure SI-1. Stability of RRM1+2 HuR protein in different buffer assessed by TSA. …SI-5
- Figure SI-2. Procedure for preparing the four reaction mixtures for pt-DCC assay. …SI-6
- Figure SI-3. HPLC-UV trace for the comparative analysis of the pt-DCC assay performed in acetate buffer. Amplified compound 2 is marked with an asterisk. …SI-8
- Figure SI-4. HPLC-UV trace for the comparative analysis of the pt-DCC assay performed in acetate buffer. Amplified compound 5 is marked with an asterisk …SI-9
- Figure SI-5. HPLC-UV trace for the comparative analysis of the pt-DCC assay performed in phosphate buffer. Amplified compound 2 is marked with an asterisk. …SI-10
- Figure SI-6. HPLC-UV trace for the comparative analysis of the pt-DCC assay performed in phosphate buffer. Amplified compound 5 is marked with an asterisk. …SI-11
- Figure SI-7. ^{1}H-NMR and STD NMR spectra for the most amplified fragments in the pt-DCC assay. …SI-13
- Figure SI-8. 2D representation of A) 7; B) 2; C) 6; D) 1; E) 3; F) 4 in complex with HuR protein. Hydrogen bonds, π-π stacking and halogen bonds are shown, respectively, as magenta, green and sand lines. …SI-18
- Figure SI-9. % of FP emission of free mRNA in presence of compounds 5 and EGCG. …SI-21

Material and Methods

Protein expression and purification

The protein expression and purification were performed as previously reported but using the coding sequences for the RRM1+2 protein (residues 1 to 186) cloned into the pETM-11 expression vector. This vector incorporates an N-terminal polyhistidine-tag (His6-tag), which precedes a tobacco etch virus (TEV) protease cleavage site. The recombinant plasmids were transformed into *Escherichia coli* BL21(DE3) competent cells. These cells were spread on lysogeny broth (LB)-agar plates containing kanamycin and left to incubate overnight at 37 °C. Thereafter, a single colony was selected and inoculated in LB medium supplemented with kanamycin and incubated overnight at the same temperature. The preculture was then diluted 1:100 with ZYM-5052 medium for auto-induction, which was further enriched with kanamycin and incubated at 37 °C. Upon the cell density (OD600) reaching 0.8, the temperature was reduced to 20 °C, and the culture was incubated overnight. The cells were then harvested via centrifugation, resuspended in lysis buffer, and lysed by sonication. After the lysate was clarified through centrifugation, the supernatant was loaded onto a Ni-NTA (nickel-nitrilotriacetic acid) agarose column (Qiagen, Germany). The column was washed to eliminate non-specifically bound products, and the target protein was eluted with an elution buffer supplemented with 250 mM imidazole. The flow-through was combined with TEV protease and dialyzed overnight at 4 °C. Both the TEV protease and cleaved His6-tag were removed by a second Ni-NTA affinity chromatography step. The concentrated eluate was ultimately purified through size-exclusion chromatography (SEC) on a Superdex 75 Hiload 16/60 column (GE Healthcare) in a 20 mM sodium phosphate buffer with pH 7.0, 200 mM NaCl, 1 mM DTT, and 1 mM EDTA. The purified proteins were concentrated, flash-frozen in liquid nitrogen, and stored at -80 °C until use. The protein purity was assessed by SDS-PAGE (sodium dodecyl sulphate-polyacrylamide gel electrophoresis), and the concentration was measured by assessing the absorbance at 280 nm. A thermal shift assay (TSA) was performed in their native buffer (20 mM Phosphate buffer pH 7.0) at six different protein concentrations (0.25–10 μM).

Establishment of experimental conditions for the pt-DCC experiment

Selection of the proper buffer for pt-DCC by TSA

A 96-well plate, holding duplicate preparations of 10 µM protein solutions in sixteen diverse buffers (Table SI-1), water, and 5% DMSO, was maintained at room temperature for a four-day span, and subjected to daily sampling (from day 0 to day 3) for TSA experimentation. The test solution was arranged as depicted in Table SI-2. Blank and negative control wells were incorporated into each TSA run.

Table SI-1. Buffer used for the assessment of pt-DCC.

Buffer Type	pH
Acetate 10 mM	5–5.5–6
MES 20 mM	5.5–6–6.5
Bis-TRIS 20 mM	5.5–6–6.5–7
Phosphate 50 mM	5.5–6–6.5–7–7.5

The dye utilized was SyproOrange (Thermo Fischer Scientific), applied at a final concentration of 4x. The plate was subjected to a thermal cycle (from 21 °C to 95 °C, with an incremental temperature ramp of 0.5°C per minute) within a real-time PCR instrument.

Table SI-2. Composition of the well for TSA analysis.

**µL protein**	2
**µL dye**	2.5
**µL buffer**	19.25
**µL DMSO**	1.25
**Total µL**	25

Melting curves were analyzed using Protein Thermal Shift 1.3 software, and the Tm recorded for each sample was plotted against time (Figure SI-1).

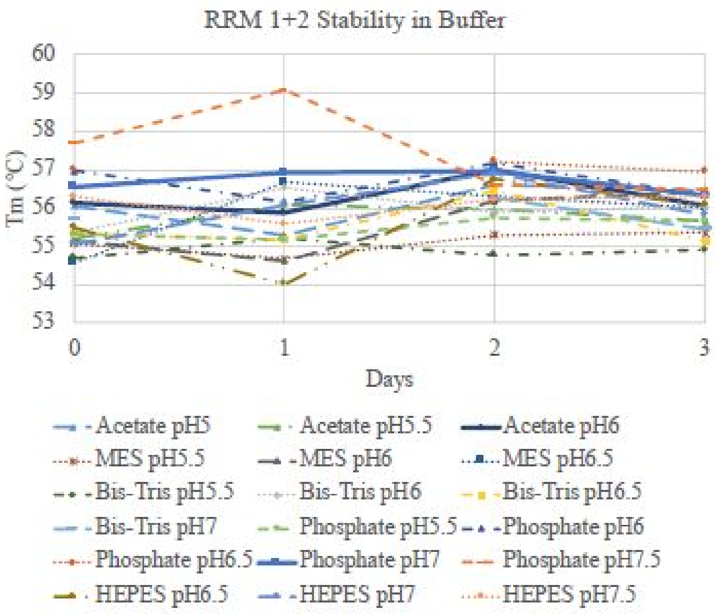

Figure SI-1. Stability of RRM1+2 HuR protein in different buffer assessed by TSA.

pt-DCC experiment

Experimental setup

The experimental procedure involved the preparation of four reaction mixtures as delineated in Figure SI-2.

Blank	Amount	Final concentration
Buffer	190 µL	-
Aldehydes		100 µM
Hydrazides	Premixed*	200 µM
Aniline	10 µL	1 mM
DMSO		5%
Total	*200 µL*	

pt-DCC	Amount	Final concentration
Buffer	126 µL	-
Aldehydes		100 µM
Hydrazides	Premixed*	200 µM
Aniline	10 µL	1 mM
DMSO		5%
RRM1+2 (125 µM)	64 µL	40 µM
Total	*200 µL*	

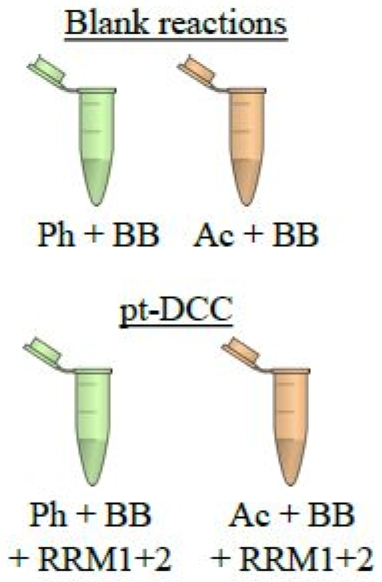

Pre-mixed solution*,#

BB+aniline in DMSO	Amount	Final concentration
Aldehydes (100 mM)	1 µLx 2	2 mM
Hydrazides (100 mM)	2 µL x 8	4 mM
Aniline (1 M)	1 µL	20 mM
DMSO	31 µL	-
Total	*50 µL*	

The preparation of the pre-mixed solution followed a precise order to avoid triggering the reaction: 1) Hydrazides; 2) Aldehydes; 3) Aniline. The premixed solution was then added last to the reaction mixture.

Figure SI-2. Procedure for preparing the four reaction mixtures for pt-DCC assay.

The reaction mixtures were stirred for 36 hours and sampled at regular intervals. For HPLC-UV-MS analysis, samples were prepared as following: 5 µL of each reaction sample were introduced into an Eppendorf tube containing 44 µL of analytical-grade ACN (to precipitate the protein) and 1 µL of 1 M NaOH (to stop the reaction reversibility). The vials were vortexed for 10 seconds and then centrifuged at 14,000 RPM for 5 minutes at 4 °C. The supernatant was collected and immediately analyzed. Previously collected samples were stored at 4 °C.

HPLC method

HPLC-UV-MS analyses were performed on a ThermoScientific Dionex Ultimate 3000 UHPLC System, connected to a ThermoScientific Q Exactive Focus equipped with an electrospray ion source. Separation was performed on an Acquity Waters Column (BEH, C8 1.7 µm, 2.1 x 150 mm, Waters, Germany) with an accompanying VanGuard Pre-Column (BEH C8, 5 x 2.1 mm, 1.7 µm, Waters, Germany). The elution was performed at a flow rate of 0.250 mL/min in gradient mode. The mobile phase consisted of solvent A (ACN + 0.1% HCOOH) and solvent B (H2O + 0.1% HCOOH). The initial mobile phase composition was maintained at 10% solvent A for 1 min, changed linearly to 95% of A in 16 min and held for 1.5 min, then followed by a return to the initial conditions within 0.1 min and kept 2 min for the chromatograph column equilibrium. The mass spectrum was recorded in positive mode within a range of 100 – 700 m/z.

Determination of Equilibrium and Product Amplification Quantification

The products within the reaction mixture were uniquely identified by high-resolution mass, and the peak area was measured on the HPLC-UV-trace at λ = 272 nm. To assess that equilibrium was reached, the area of each peak in blank sample was plotted against time. Once the area reaches a value stable in time, the equilibrium was considered reached and the amplification was investigated in pt-DCC samples. Amplification was calculated for each peak by attributing an arbitrary value of 100% to the area in the blank, following the proportion:

AreaDCC: x = Areablank : 100. Then, x = (AreaDCC * 100) / Area blank.

The amplification % is then calculated as A% = x – 100.

There is no established cut-off for the selection of amplified products, as this is contingent on the quantity of protein employed in the assay and on the product's affinity for the protein, low A% values might typically be ascribed to slight integration differences rather than actual amplification.

Comparative Analysis

Acetate Buffer

For compound **2**, the equilibrium was reached after 10h. The amplification was measured at 10, 24 and 30 h, resulting in 83% (at 10h) and 107% (at 24h).

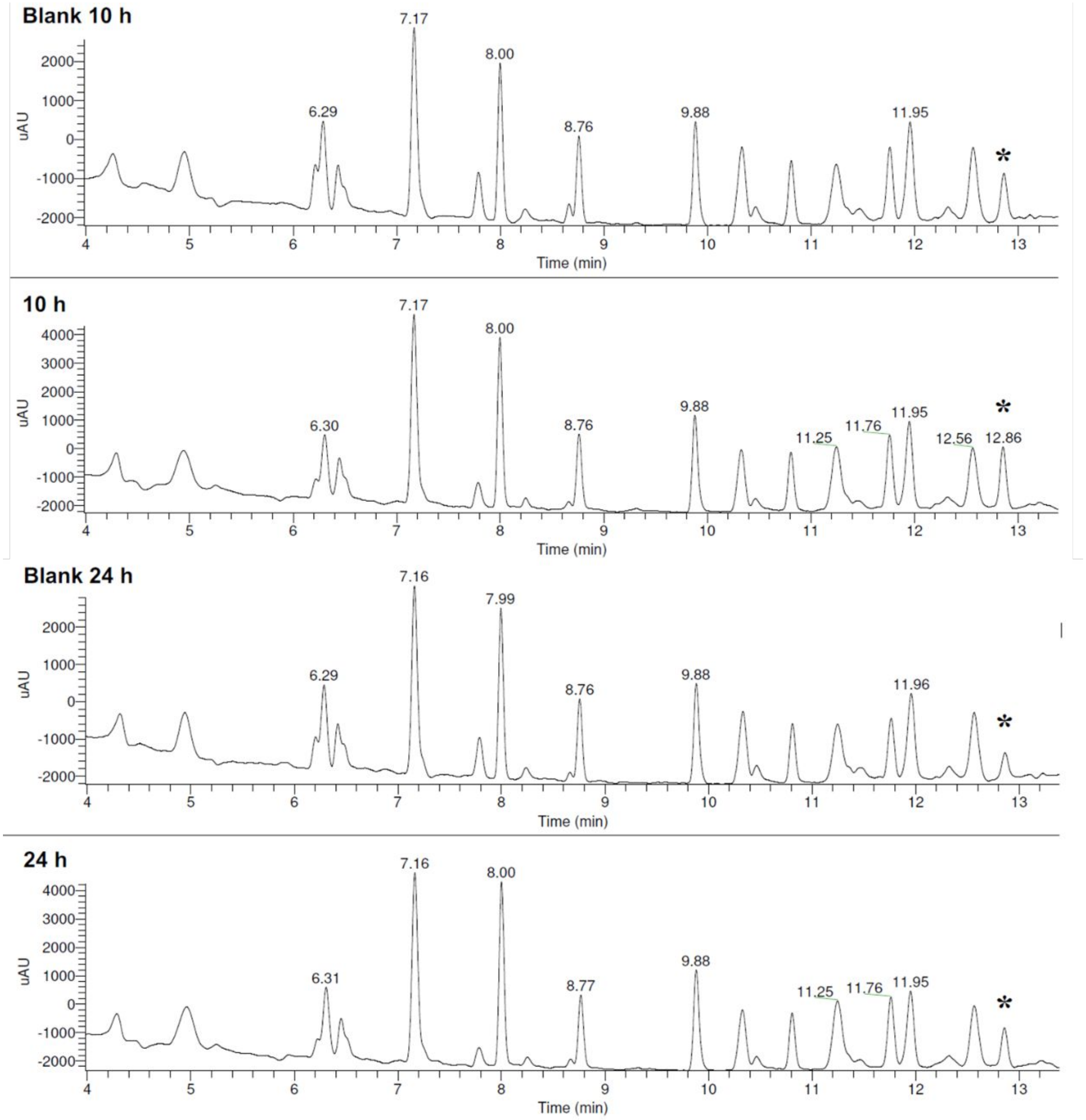

Figure SI-3. HPLC-UV trace for the comparative analysis of the pt-DCC assay performed in acetate buffer for compound **2** (marked with an asterisk).

For compound **5**, the equilibrium was reached after 10h. The amplification was measured at 10, 24 and 30 h, resulting in 121% (at 10h).

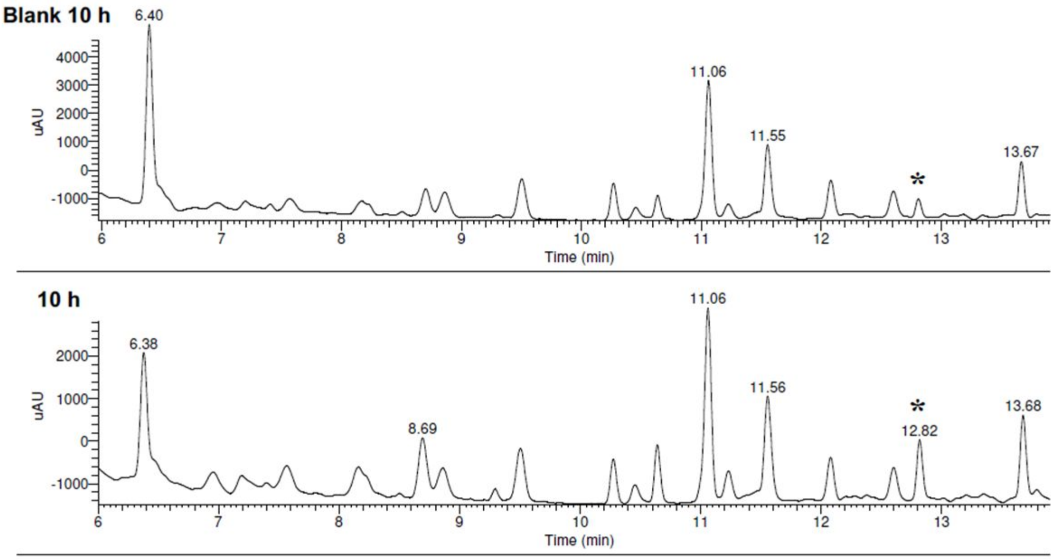

Figure SI-4. HPLC-UV trace for the comparative analysis of the pt-DCC assay performed in acetate buffer for compound **5** (marked with an asterisk).

Phosphate Buffer

For compound **2**, the equilibrium was reached after 12h. The amplification was measured at 12, 24 and 30 h, and significant values were found at 10 and 24 h with an amplification of 121% and 328% (24h), respectively.

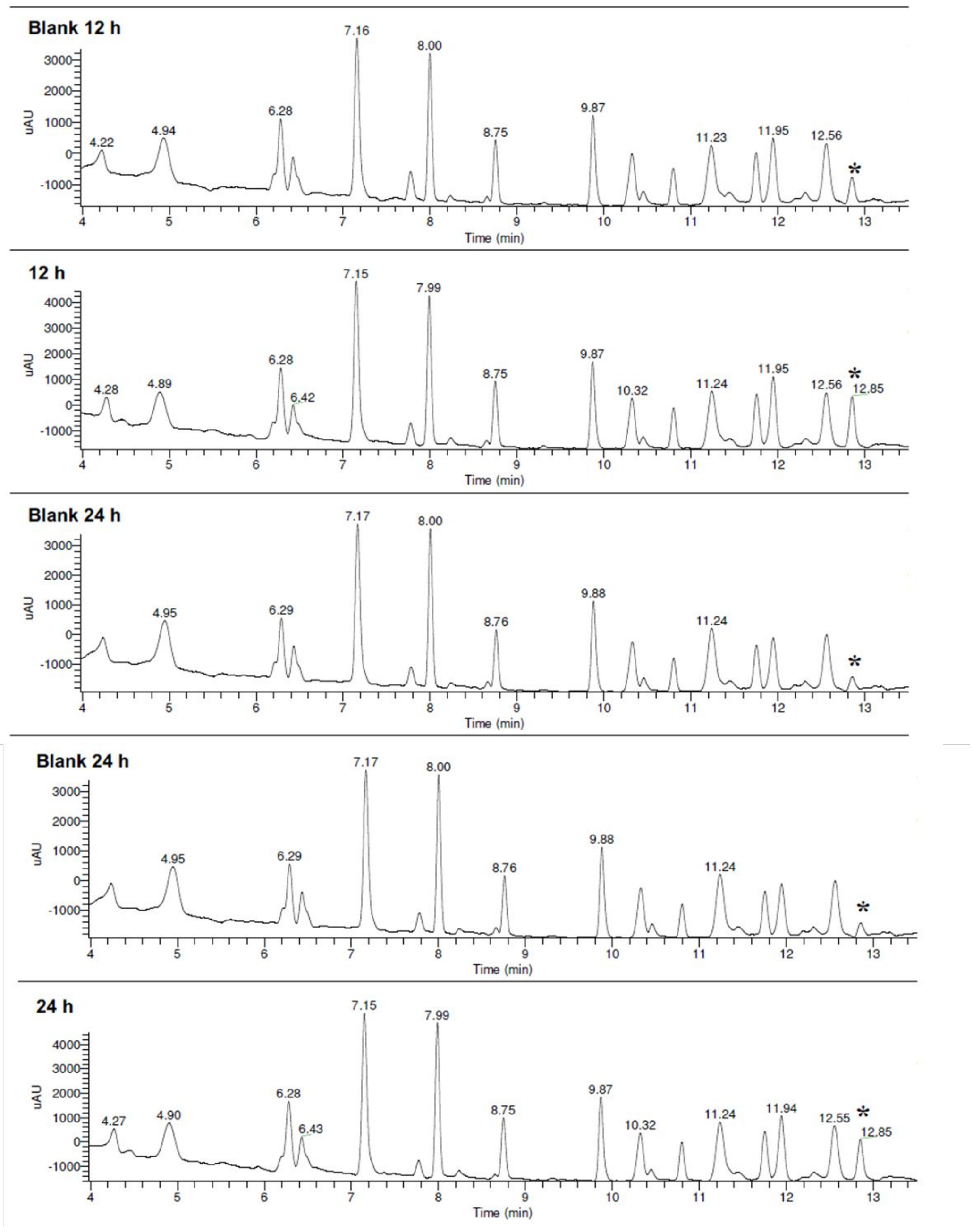

Figure SI-5. HPLC-UV trace for the comparative analysis of the pt-DCC assay performed in phosphate buffer for compound **2** is marked with an asterisk.

For compound **5**, the equilibrium was reached after 10h. The amplification was measured at 10, 24 and 30 h, and significant values were found at 10 with an amplification of 89%.

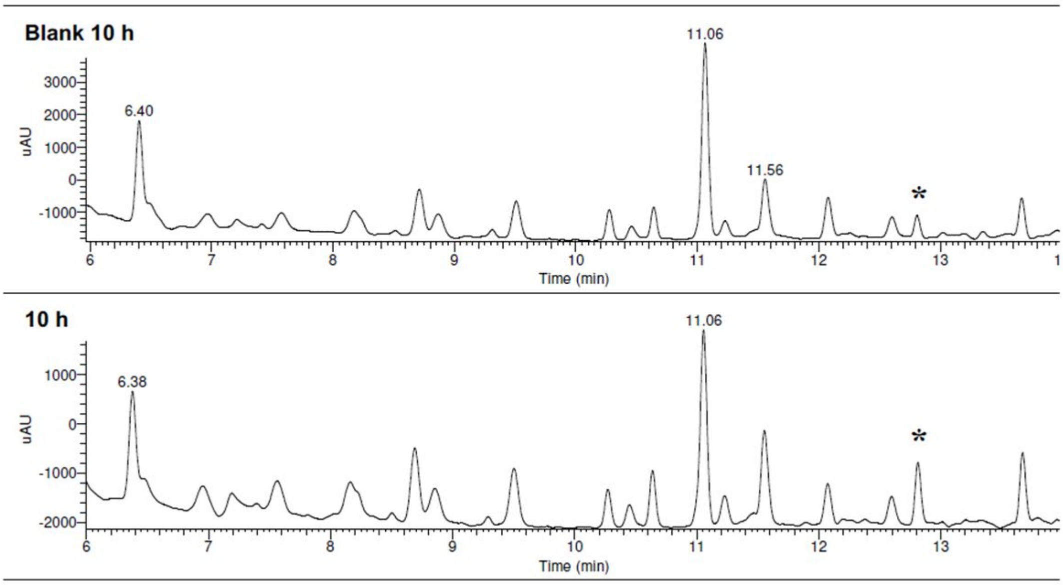

Figure SI-6. HPLC-UV trace for the comparative analysis of the pt-DCC assay performed in phosphate buffer for compound **5** (marked with an asterisk).

STD-NMR assays

All protein−ligand samples were prepared in a 1000:1 ligand/protein ratio. Typically, the final concentration of the samples was 400 μM of ligand and 0.4 μM of HuR, and the final volume was 200 μL. The buffer used was a 20 μM deuterated phosphate buffer pH 7.4.

^{1}H-STD-NMR experiments were performed on a 600 MHz Bruker Avance spectrometer. The probe temperature was maintained at 283 K. In the STD experiments, water suppression was achieved by the WATERGATE 3-9-19 pulse sequence. The on-resonance irradiation of the protein was performed at −0.05 ppm. Off-resonance irradiation was applied at 200 ppm, where no protein signals are visible. Selective presaturation of the protein was achieved by a train of Gauss-shaped pulses of 49 ms length each. The STD-NMR spectra were acquired with an optimized total length of saturation train of 2.94 s. Blank experiments were conducted in absence of protein in order to avoid artefacts.

Intensities of all STD effects (absolute STD) were calculated by division through integrals over the respective signals in STD-NMR reference spectra. The different signal intensities of the individual protons are best analyzed from the integral values in the reference and STD spectra, respectively. $(I0-I_{sat})/I0$ is the fractional STD effect, expressing the signal intensity in the STD spectrum as a fraction of the intensity of an unsaturated reference spectrum. In this equation, I0 is the intensity of one signal in the off-resonance or reference NMR spectrum, I_{sat} is the intensity of a signal in the on-resonance NMR spectrum, and I0-Isat represents the intensity of the STD-NMR spectrum[1–3].

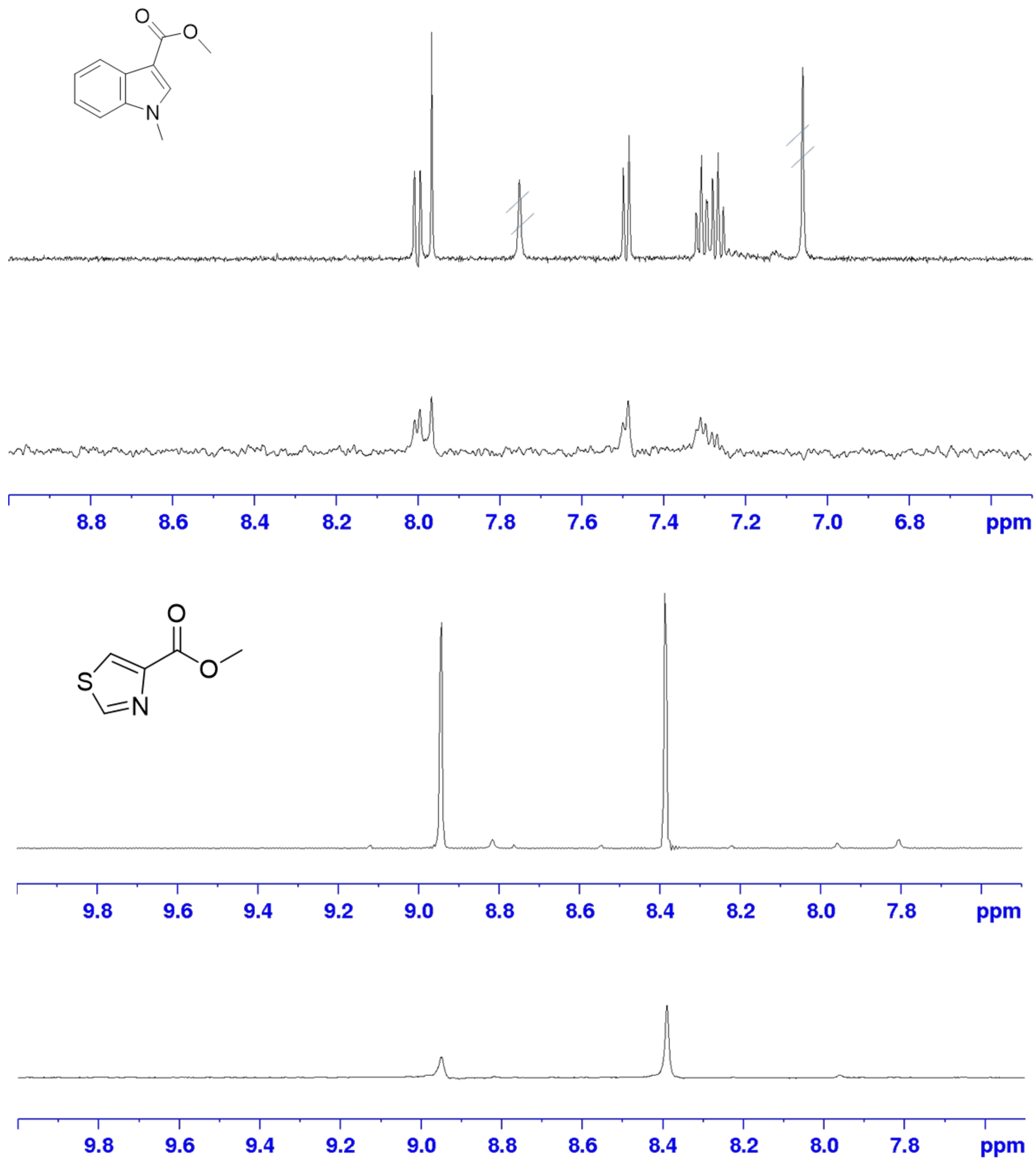

Figure SI-7. ^{1}H-NMR and STD NMR spectra for the most amplified fragments in the pt-DCC assay.

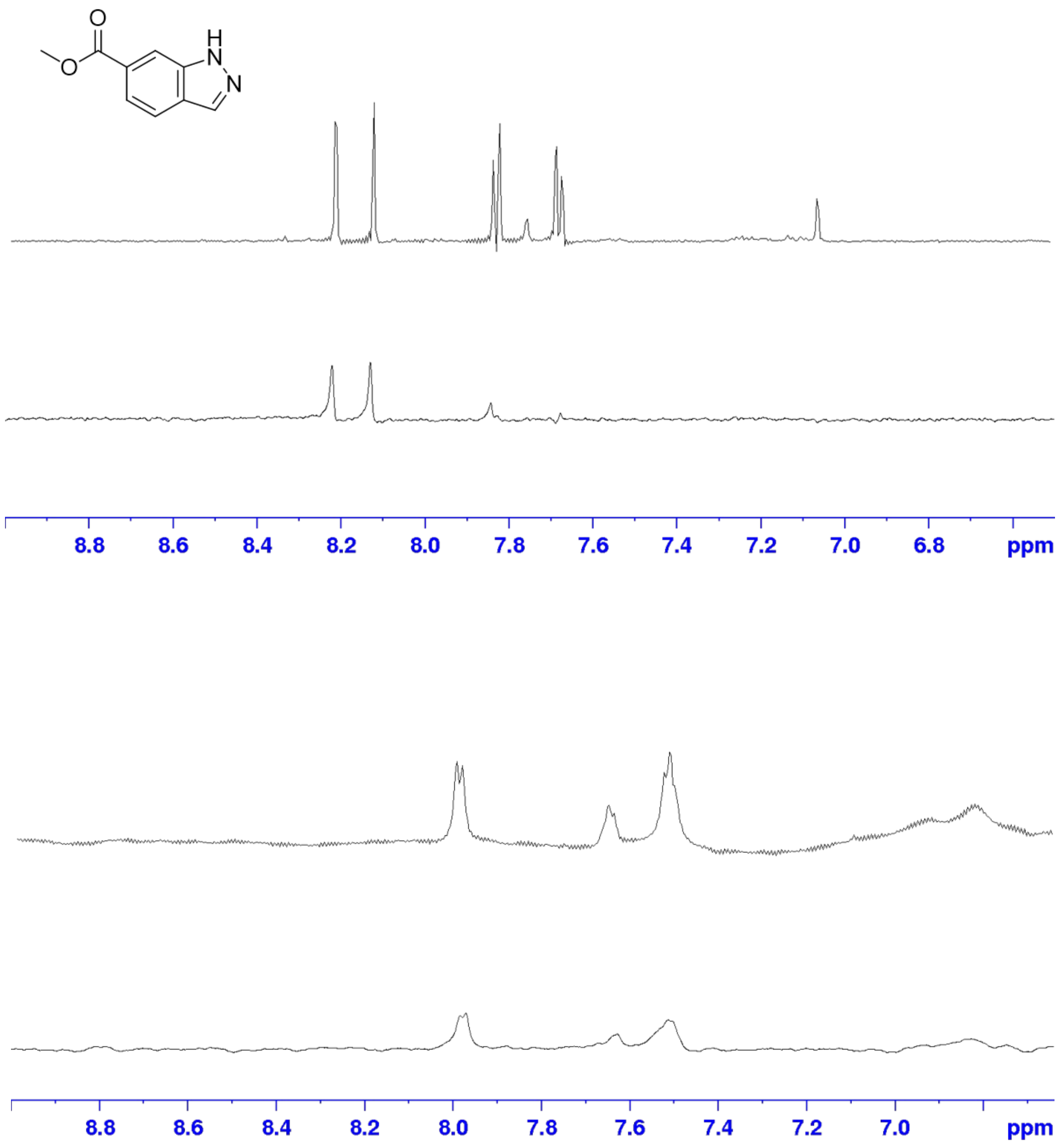

Figure SI-7. (segue)

Chemistry

General procedure for the synthesis of acylhydrazones

An equimolar amount of aldehyde (approximately 50 mg) and hydrazide were dissolved in dry methanol under a nitrogen atmosphere, after which the reaction mixture was heated to reflux and stirred overnight. The final compounds typically precipitated as white or yellowish solids. These solids were rinsed with cold methanol (3 x 3 mL) and subjected to centrifugation thrice. The remaining solvent was subsequently evaporated under reduced pressure. The purity and retention time (Rt) were verified via HPLC, and the identity of the compounds was confirmed through HRMS and ^{1}H NMR. Average yield: 65-80%.

Characterization of synthesized compounds

^{1}H NMR spectra were acquired in either DMSO-d6 (D) or $CDCl_3$ (Cl), utilizing a Bruker Fourier 500 spectrometer (500 MHz). The chemical shifts are documented in parts per million (ppm), relative to the pertinent solvent peak. The coupling constants associated with splitting patterns are reported in Hz and categorized as singlet (s), doublet (d), triplet (t), or multiplet (m). ^{1}H-NMR attribution for the seven synthesized acylhydrazones are reported in Table SI-3. Owing to the presence of isomers for acylhydrazones, some signals may appear duplicated (minor form denoted in grey in Table SI-3).

Table SI-3. ^{1}H-NMR characterization for compounds **1-7**.

Cmpd	Structure	^{1}H-NMR (ppm)[a]
**1**	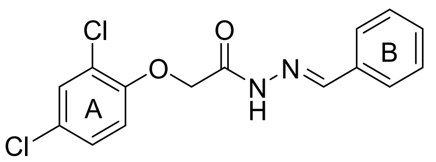	Isomer I: 11.69 (N*H*, s); 8.01 (C*H*, s); 7.72 (2H, 2,6 B, o/l); 7.59 (1H, 3 A s); 7.45 (3H, 3,4,5 B, d, J = 5.84); 7.34 (1H, 5 A, d, J = 9.35); 7.09 (1H, 6 A d, J = 9.35); 5.32 (2H, C*H2*, s). Isomer II: 11.65 (N*H*, s); 8.27 (C*H*, s); 7.71 (2H, 2,6 B, o/l); 7.63 (1H, 3 A, s); 7.45 (3H, 3,4,5 B, o/l); 7.40 (1H, 5 A, d, J = 9.35); 7.12 (1H, 6 A d, J = 9.35); 4.82 (2H, C*H2*, s). ratio ~3:1
**2**	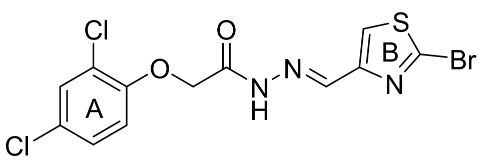	Isomer I: 11.78 (N*H*, s); 8.16 (5 B, s); 8.04(C*H*, s); 7.59 (1H, 3 A, d, J = 2.4); 7.33 (1H, 5 A, dd, J = 2.4; 8.7); 7.06 (1H, 6 A, d, J = 8.7); 5.27 (CH2, s). Isomer II: 11.75 (N*H*, s); 8.28 (5 B, s); 8.12 (C*H*, s); 7.62 (1H, 3 A, d, J = 2.4); 7.41 (1H, 5 A, dd, J = 2.4; 8.7); 7.1 (1H, 6 A, d, J = 8.7); 4.84 (CH2, s). ratio ~3:1.
**3**	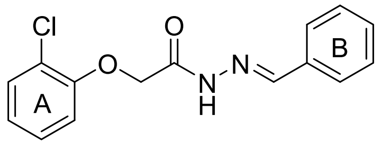	Isomer I: 11.64 (N*H*, s); 8.02 (C*H*, s); 7.72 (2H, 2,6 B, o/l); 7.45 (4H, 3,4,5 B, o/l; 3 A o/l); 7.27 (1H, 5 A, t, J = 7.72); 7.03 (1H, 6 A d, J = 8.37); 6.96 (1H, 4 A, t, J = 7.72); 5.32 (2H, C*H2*, s). Isomer II: 11.67 (N*H*, s); 8.28 (C*H*, s); 7.73 (2H, 2,6 B, o/l); 7.45 (4H, 3,4,5 B, o/l; 3 A o/l); 7.31 (1H, 5 A, t, J = 7.72); 7.09 (1H, 6 A d, J = 8.37); 7.01 (1H, 4 A, t, J = 7.72); 4.82 (2H, C*H2*, s). ratio ~2.5:1
**4**	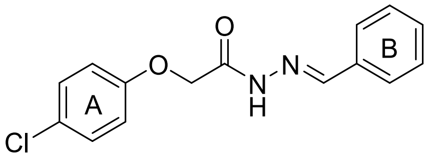	Isomer I: 11.64 (N*H*, s); 8.02 (C*H*, s); 7.71-7.45 (5H, 3,4,5 B; 2,6 B, o/l); 7.33 (2H, 3,5 A, dt); 6.97 (2H, 2,6 A dt); 5.17 (2H, C*H2*, s). Isomer II: 11.59 (N*H*, s); 8.34 (C*H*, s); 7.71-7.45 (5H, 3,4,5 B; 2,6 B, o/l); 7.38 (2H, 3,5 A, dt); 7.03 (2H, 2, A dt); 4.69 (2H, C*H2*, s). ratio ~1.8:1

**5**	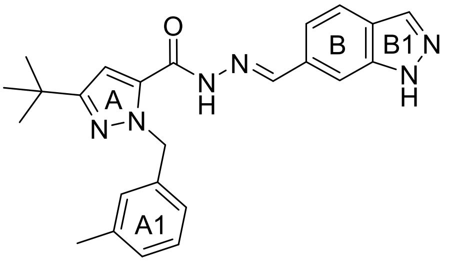	13.28 (N*H* B1, s); 11.84 (N*H*, s); 8.50 (C*H*, s); 8.13 (1H, 2 B, d); 7.83 (1H, 5 B, d, J = 8.31); 7.79 (1H, 3 B1, s); 7.56 (1H, 6 B d, J = 8.31); 7.19 (1H, 5 A1, t, J = 7.72); 7.06 (1H, 6 A1, d, J = 7.72); 6.98 (1H, 2 A1 s); 6.96 (1H, 5 A s); 6.90 (1H, 4 A1, d, J = 7.72); 5.68 (2H, C*H2*, s); 2.28 (3H, C*H3*, s), 1.31 (9H, (C*H3*)*3*, s).
**6**	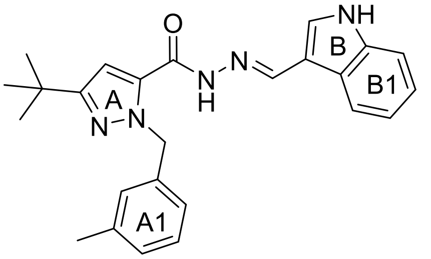	Isomer I: 11.61 (N*H,* B, s); 11.48 (N*H*, s, o/l); 8.55 (1H, s); 8.26 (1H, d); 7.83 (1H, s); 7.44 (1H, d); 7.23–7.13 (3H, m; o/l); 7.06–6.98 (2H, m; o/l); 6.94–6.90 (2H, m; o/l); 5.70 (CH2, s); 2.26 (CH3, s); 1.31 ((CH3)3, s);<br>Isomer II: 11.48 (N*H*, s, o/l); 8.07 (1H, d); 7.81 (1H, s); 7.23–7.13 (3H, m; o/l); 7.06–6.98 (2H, m; o/l); 6.94–6.90 (2H, m; o/l); 5.63 ((CH3)3, s); 2.17 (CH3, s); 1.35 ((CH3)3, s); ratio ~5:1 (full assignment pending 2D spectra due to overlap)
**7**	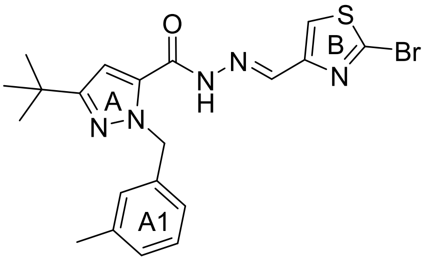	13.19 (N*H*, s); 7.53 (1H, 5 B, s); 7.32 (C*H*, s); 7.15 (1H, 2 A1, s); 7.09 (2H, 4,6 A1, d, J = 6.93); 6.96 (1H, d, 5 A1, J = 6.93); 6.64 (1H, 5 A, s); 5.74 (C*H*2, s); 2.23 (C*H*3, s); 1.31 ((C*H*3)3, s).

a. o/l = overlap of peak chemical shift in the ^{1}H NMR spectrum

Molecular modeling

Molecular modeling simulations were carried out starting from the crystal structure of the two N-terminal RRM domains of HuR complexed with RNA, deposited in the Protein Data Bank (PDB) with the PDB code 4ED5 [4]

The preparation of the complex and the choice of the best HuR protein conformation, then used for the further computational studies, were carried out according to the procedure reported in our previously published works.[1–3]

The N-acylhydrazone derivatives were prepared by means of LigPrep tools, at pH 7.4, using OPLS_2005 as force field.[5]

Docking simulations were carried out with Glide software v. 7.8 by using the standard precision (SP) algorithm, and 10 poses for ligand were generated. [6]

Then, for each compound the best docking pose was subjected to Molecular Mechanism Generalized Born Suface Area (MM-GBSA), using VSGB as solvation model and OPLS_2005 as force field. [7,8]

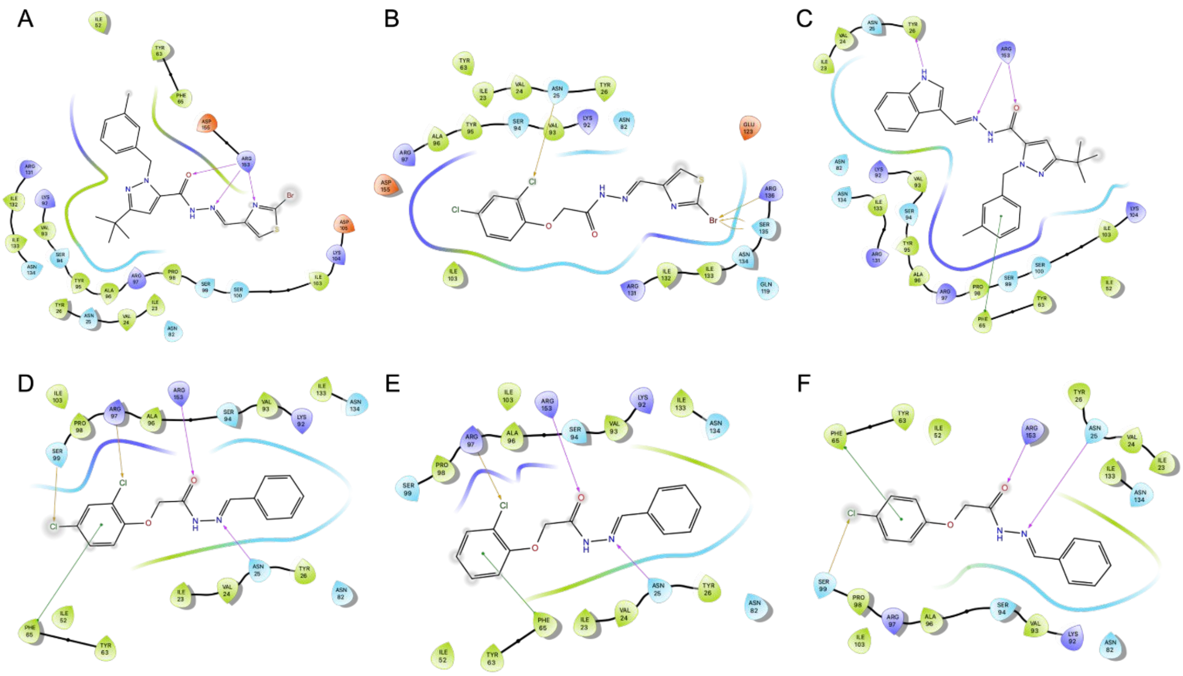

Figure SI-8. 2D representation of A) **7**; B) **2**; C) **6**; D) **1**; E) **3**; F) **4** in complex with HuR protein. Hydrogen bonds, π-π stacking and halogen bonds are shown, respectively, as magenta, green and sand lines. The 2D representation was built after the MM-GBSA post-docking analysis.

Fluorescence polarization assays

As previously reported, the sequence of the fluorescently labeled (5′ -FAM) RNA probe is UAUUUAUUUA. Fluorescence polarization assays were performed in PBS (20 mM sodium phosphate, 150 mM NaCl, 1 mM dithiothreitol, 0.05% PLURONIC-127, 15% DMSO) at 293 K using a SpectraMax M5 microplate-reader system. The assay was optimized first by titrating both HuR with fluorescently labeled mRNA[9]. The wavelengths of fluorescence excitation and emission were 490 and 522 nm, respectively. Based on the fluorescence emission, 2.4 µM of HuR, and 45 nM of labeled mRNA were used for the assay. The binary complex HuR–mRNA showed a basal FP emission of 105 mP, whereas the labeled mRNA alone displays a residual FP emission of 35 mP, in the same experimental conditions. Each well of a 384-well plate contained 45 nM 5′-FAM RNA probe and 2.4 uM of HuR RRM1/2. For each assay, rutin as positive control and usnic acid as negative control were included. In the compound competition assay, compounds with eight doses (500 nM–100 µM) were added to the protein The compounds were added to the protein, and the system was incubated for 1 h to allow the assessment of the binding of the compounds with HuR. Thereafter, the mRNA was added and, after another incubation for 1.5 h, the FP emission of the binary HuR–RNA complex was acquired. The percent of complexation was calculated by comparison to a DMSO control. The FP value of the HuR–RNA complex with DMSO was defined as 100% of complexation; the FP value of labeled RNA only was defined as 0% of complexation. IC_{50}, the concentration causing 50% of the dissociation of the HuR-mRNA complex, was extrapolated via sigmoidal fitting of the dose–response curve using Prism 5.0. The experiments were performed in duplicate and SD is ±10%.

Table SI-4. Percentage of residual protein polarization at 100 μM concentration of the tested compounds

Cmpd	% of residual protein polarization @100 μM
**1**	82%
**2**	75%
**3**	85%
**4**	80%
**5**	59%
**6**	76%
**7**	68%

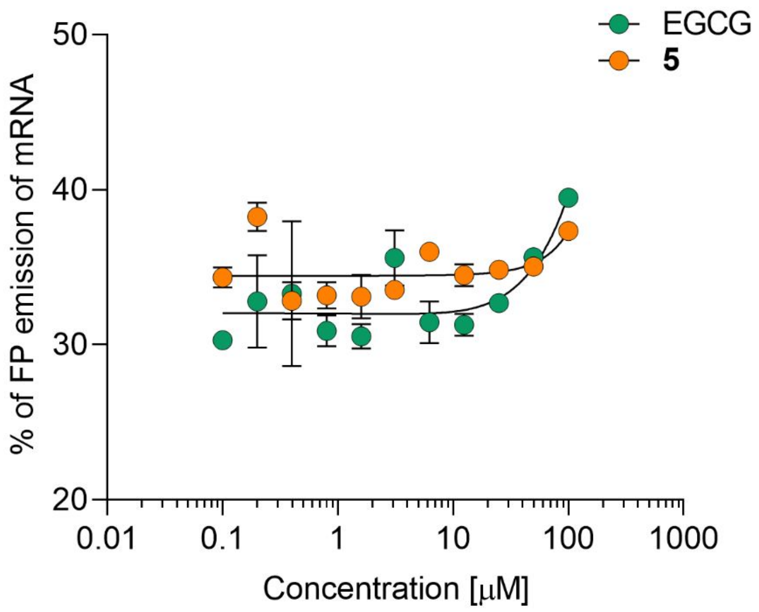

Figure SI-9. % of FP emission of free mRNA in presence of compounds **5** and EGCG.

REFERENCES

(1) Della Volpe, S.; Nasti, R.; Queirolo, M.; Unver, M. Y.; Jumde, V. K.; Dömling, A.; Vasile, F.; Potenza, D.; Ambrosio, F. A.; Costa, G.; Alcaro, S.; Zucal, C.; Provenzani, A.; Di Giacomo, M.; Rossi, D.; Hirsch, A. K. H.; Collina, S. Novel Compounds Targeting the RNA-Binding Protein HuR. Structure-Based Design, Synthesis, and Interaction Studies. *ACS Med. Chem. Lett.* **2019**, *10* (4), 615–620. https://doi.org/10.1021/acsmedchemlett.8b00600.

(2) Volpe, S. D.; Listro, R.; Parafioriti, M.; Di Giacomo, M.; Rossi, D.; Ambrosio, F. A.; Costa, G.; Alcaro, S.; Ortuso, F.; Hirsch, A. K. H.; Vasile, F.; Collina, S. BOPC1 Enantiomers Preparation and HuR Interaction Study. From Molecular Modeling to a Curious DEEP-STD NMR Application. *ACS Med. Chem. Lett.* **2020**, *11* (5), 883–888. https://doi.org/10.1021/acsmedchemlett.9b00659.

(3) Vasile, F.; Volpe, S. D.; Ambrosio, F. A.; Costa, G.; Unver, M. Y.; Zucal, C.; Rossi, D.; Martino, E.; Provenzani, A.; Hirsch, A. K. H.; Alcaro, S.; Potenza, D.; Collina, S. Exploration of Ligand Binding Modes towards the Identification of Compounds Targeting HuR: A Combined STD-NMR and Molecular Modelling Approach. *Sci Rep* **2018**, *8* (1), 1–11. https://doi.org/10.1038/s41598-018-32084-z.

(4) Wang, H.; Zeng, F.; Liu, Q.; Liu, H.; Liu, Z.; Niu, L.; Teng, M.; Li, X. The Structure of the ARE-Binding Domains of Hu Antigen R (HuR) Undergoes Conformational Changes during RNA Binding. *Acta Cryst D* **2013**, *69* (3), 373–380. https://doi.org/10.1107/S0907444912047828.

(5) LigPrep, Schrödinger, SiteMap, LLC. **2018**.

(6) Glide, Schrödinger, SiteMap, LLC. **2018**.

(7) Prime, Schrödinger, SiteMap, LLC. **2018**.

(8) Jorgensen, W. L.; Maxwell, D. S.; Tirado-Rives, J. Development and Testing of the OPLS All-Atom Force Field on Conformational Energetics and Properties of Organic Liquids. *J. Am. Chem. Soc.* **1996**, *118* (45), 11225–11236. https://doi.org/10.1021/ja9621760.

(9) Della Volpe, S.; Linciano, P.; Listro, R.; Tumminelli, E.; Amadio, M.; Bonomo, I.; Elgaher, W. A. M.; Adam, S.; Hirsch, A. K. H.; Boeckler, F. M.; Vasile, F.; Rossi, D.; Collina, S. Identification of N,N-Arylalkyl-Picolinamide Derivatives Targeting the RNA-Binding Protein HuR, by Combining Biophysical Fragment-Screening and Molecular Hybridization. *Bioorganic Chemistry* **2021**, *116*, 105305. https://doi.org/10.1016/j.bioorg.2021.105305.
